# The V617I Substitution in Avian Coronavirus IBV Spike Protein Plays a Crucial Role in Adaptation to Primary Chicken Kidney Cells

**DOI:** 10.3389/fmicb.2020.604335

**Published:** 2020-12-18

**Authors:** Yi Jiang, Mingyan Gao, Xu Cheng, Yan Yu, Xinyue Shen, Jianmei Li, Sheng Zhou

**Affiliations:** ^1^Poultry Institute, Chinese Academy of Agricultural Sciences, Yangzhou, China; ^2^Jiangsu Institute of Poultry Science (CAAS), Yangzhou, China; ^3^Laboratory of Animal Infectious Disease, College of Veterinary Medicine, Yangzhou University, Yangzhou, China

**Keywords:** infectious bronchitis virus, CK cells, tropism, amino acid mutation, spike

## Abstract

The naturally isolated avian coronavirus infectious bronchitis virus (IBV) generally cannot replicate in chicken kidney (CK) cells. To explore the molecular mechanism of IBV adapting to CK cells, a series of recombinant viruses were constructed by chimerizing the S genes of CK cell-adapted strain H120 and non-adapted strain IBYZ. The results showed that the S2 subunit determines the difference in cell tropism of the two strains. After comparing the amino acid sequences of S protein of CK cell-adapted strain YZ120, with its parental strain IBYZ, three amino acid substitutions, A138V, L581F, and V617I, were identified. Using YZ120 as the backbone, one or more of the above-mentioned substitutions were eliminated to verify the correlation between these sites and CK cell tropism. The results showed that the CK cell tropism of the YZ120 strain depends on the V617I substitution, the change of L581F promoted the adaptation in CK cells, and the change at 138 position was not directly related to the CK cell tropism. Further validation experiments also showed that V617I had a decisive role in the adaptation of IBV to CK cells, but other areas of the virus genome also affected the replication efficiency of the virus in CK cells.

## Introduction

Avian coronavirus IBV is a positive-sense RNA enveloped virus, which belongs to the order Nidovirales, family Coronaviridae, genus Gammacoronavirus (Cavanagh, 2007). Like other coronaviruses (CoVs), IBV consists of basic structural proteins, including spike (S), membrane (M), nucleocapsid (N), envelope (E) protein, and the genome encodes other accessory and non-structural proteins. The CoV spike protein is a class I fusion protein (Bosch et al., 2003), the ectodomain consists of S1 and S2 domains, and it plays a major role in the process of viral infection (Cavanagh, 1983; Belouzard et al., 2012). The S1 subunit contains the receptor-binding domain (RBD) located on the N-terminal domain (NTD) or C-terminal domain (CTD), which specifically binds the host receptors, such as a variety of proteins and sugars (Belouzard et al., 2012; Li, 2012). The S2 subunit, responsible for virus-cell and cell-cell fusion, contains fusion peptides (FP), two heptad repeat HR1 and HR2, transmembrane span (TM), and cytoplasmic tail (CT) (Heald-Sargent and Gallagher, 2012).

Although CoVs primarily infect the respiratory and gastrointestinal tracts of a wide range of animal species, such as bats, civet cats, camels, humans, swine, chickens, turkeys, etc. (Hulswit et al., 2016), different CoVs or different serotypes of strains from the same CoV have a restricted tissue and cell tropism, which is largely determined by the spike protein (Graham and Baric, 2010; Belouzard et al., 2012). CoVs enter the cells via the plasma membrane pathway or endosomal pathway (Millet and Whittaker, 2015). Substitution of S protein may lead to changes in the cell or tissue tropism of CoVs (Kuo et al., 2000). During the infection process, the CoV spike protein can be activated and cleaved into S1 and S2 subunits at different cleavage sites by various host proteases (Belouzard et al., 2012; Xia et al., 2018). The S1 subunit is involved in the host receptor recognition, whereas the S2 subunit anchored in the virus membrane is important for membrane fusion. Only a few amino acid alterations in the RBD of the S1 subunit can change the host species tropism of the CoV (Krempl et al., 1997; Li et al., 2005; Qu et al., 2005). Four amino acid substitutions in the S2 subunit of MHV-A59 can extend the host range in the non-permissive mammalian cell types of this virus, which indicates that the S2 subunit also plays an essential role in the viral cell and tissue tropism (Baric et al., 1999; McRoy and Baric, 2008). In addition, changes in the proteolytic cleavage sites in the S protein, located at the S1/S2 boundary or immediately upstream of the FP, are associated with altered tropism. The introduction of two single amino acid substitutions (S746R and N762A) at the S1/S2 boundary of the S protein of the bat coronavirus HKU4 were found to be crucial for the adaptation to human cells (Yang et al., 2015). In addition, PEDV (porcine epidemic diarrhea virus) acquires the ability of replication in non-enteric tissues by a single-amino acid substitution at the S2’ cleavage site (Li, 2015).

Infectious bronchitis virus shows strict cell and tissue tropism. Almost all IBV strains cannot infect mammalian cell lines, with the exception of the Beaudette strain, which was generated by several hundred passages in embryonated eggs (Bickerton et al., 2018b). A large number of newly isolated strains from diseased animals do not have the ability to replicate in primary chick kidney (CK) cells and need to be passaged in embryos for adaptation (Fang et al., 2005). The spike protein has been demonstrated to be a determinant of cell tropism by a recombinant strain BeauR-M41(S), which is based on the genome backbone of Beaudette strain and with the replacement of the ectodomain of the S protein with that of M41 strain. The rescued strain acquired the cell tropism of M41, and lost the ability to replicate in Vero, BHK-21, and CEF cells (Casais et al., 2003). In the infection process, the IBV S protein is cleaved into S1 and S2 subunits at the S1/S2 cleavage site. Sialic acid is recognized as the receptor bound by the IBV S1 subunits (Schultze et al., 1992; Winter et al., 2006, 2008), specific amino acids in more than one part of the QX-RBD S1 protein are required to establish binding to kidney tissue (Bouwman et al., 2020). However, when the S1 subunit of the Beaudette strain was replaced with that of the H120 or M41 strain, the recombinant strains retained the ability to replicate in Vero cells, indicating that in this case the cell tropism of IBV is determined by the S2 subunit (Wei et al., 2014; Bickerton et al., 2018a,b). In further studies, the lack of the S2’ cleavage motif was shown to result in the loss of the ability of the Beaudette strain to replicate in Vero cells (Bickerton et al., 2018a,b), and adding a furin S2’ cleavage site to a QX-type IBV, resulted in a virus with neurotropism (Cheng et al., 2019). In a previous study, we constructed two recombinant strains, rH120-S/YZ and rIBYZ-S/H120, which express the heterologous S gene of the CK cell adapted strain H120 or non-adapted strain IBYZ along with the backbone genome. The results showed that the S gene replacement with the corresponding sequence of CK cell non-adapted strain leads to the loss of replication in CK cells, while replacement with one of the adapted strains would provide the infection ability, demonstrating the S protein to be a determinant of CK cell tropism of IBVs (Jiang et al., 2017). In the present study, we constructed several recombinant strains expressing different regions of the S gene from the IBYZ strain, and with the background genome of H120 strain, which aimed to confirm that S1, S2, or S1/S2 cleavage site is involved in the determination of tropism extended to CK cells. Additional studies used the same method of mutant construction and explored the correlation between the tropism extended to CK cells and the mutation sites on S gene. Consequently, the S amino acid sequences of the CK cell adapted YZ120 strain and its parental strain IBYZ were aligned and analyzed to identify the relevant amino acid residues in the S gene that were modified in different genome backbones of H120, IBYZ, and H120-S/YZ strains; also, their importance for CK cell tropism was determined.

## Materials and Methods

### Virus Strains

**FIGURE 1:**
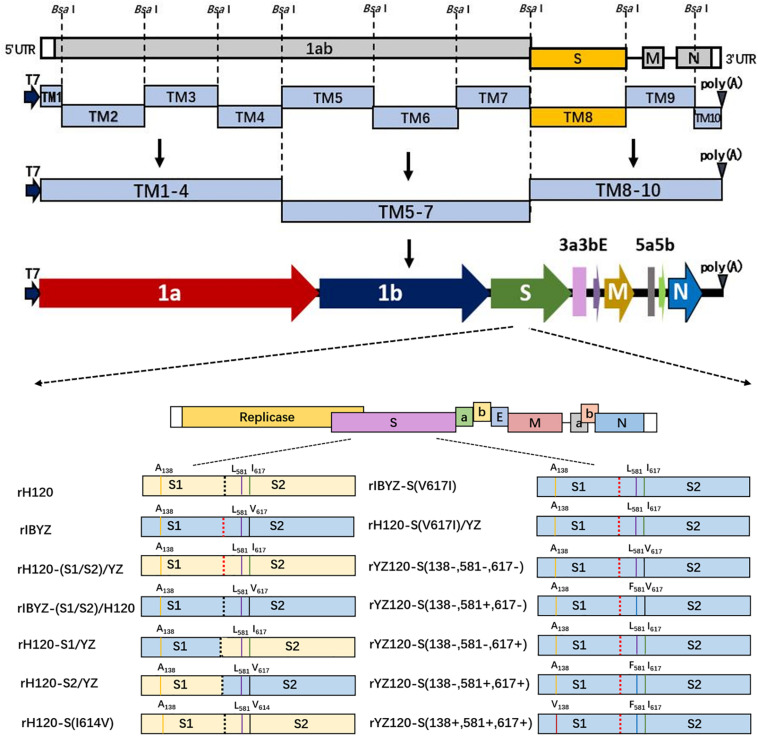
Construction of recombinant viruses with different mutant S genes. Ten cDNA fragments covering the entire viral genome were amplified by RT-PCR, and unique *Bsa* I restriction sites were introduced upstream and downstream of each cloned fragment. A unique T7 RNA polymerase promoter sequence was inserted into the 5′ end of TM1 fragment, and a 28-nucleotide A tail was introduced into the 3′ end of TM10 fragment. The original S gene fragment was replaced by the introduced mutant S gene, and the 10 fragments were sequentially connected *in vitro* with the help of appropriate ligation strategies to assemble a full-length genomic cDNA containing the mutant S gene.

The IBV strains used were (i) rH120, a molecular clone of vaccine H120 strain, which is a widely used vaccine strain in the market at present; (ii) rIBYZ, a molecular clone of strain ck/CH/IBYZ/2011 (GenBank KF663561.1), which was isolated from an IBV infected flock by our lab in Jiangsu Province, China in 2011; (iii) rYZ120 (hereafter referred to as rYZ120-S (138+, 581+, 617+), a molecular clone of CK cell adapted strain YZ120, which is derived from rIBYZ strain after multiple passages in chicken embryo (Figure 1); (iv) rH120-(S1/S2)/YZ, rIBYZ-(S1/S2)/H120, expressing the reciprocal S1/S2 cleavage site in rH120 or rIBYZ strains; (v) rH120-S1/YZ, rH120-S2/YZ, expressing chimeric S proteins composed of either the S1 subunit derived from IBYZ strain and S2 subunit from H120 or the S1 subunit derived from H120 and the S2 from IBYZ strain, with the backbone genome of H120 strain; (vi) rYZ120-S (138−, 581−, 617−), rYZ120-S (138−, 581+, 617−), rYZ120-S (138−, 581−, 617+), and rYZ120-S (138−, 581+, 617+), recombinants in which one or more amino acid sites at positions 138, 581, and 617 of the S protein were changed; (vii) rH120-S(I614V), in which the amino acid isoleucine at position 614 of the S protein was changed to valine; (viii) rIBYZ-S (V617I) and rH120-S (V617I)/YZ, expressing isoleucine at position 617 of the S protein of IBYZ, with the backbone genome of rIBYZ or rH120 strain.

### Construction of IBV Recombinant Strains

The plasmids pH120S, pIBYZS, and pYZ120S harbored the inserted S gene of the H120 vaccine strain, IBYZ strain, and YZ120 strain, respectively, which were constructed during the establishment of the reverse genetic system. By overlapping PCR technology, the furin cleavage site of S1/S2 protein of H120 and IBYZ strains were cross-replaced to construct recombinant plasmids pYZ (S1/S2)/H120 and pH120(S1/S2)/YZ. Using In-Fusion PCR cloning system (Clontech, United States), the S1 or S2 gene of the H120 strain was replaced with the corresponding region of the IBYZ strain to construct the recombinant plasmids pH120S1YZS2 and pYZS1H120S2, which contained the chimeric S genes. By overlapping PCR technology, point mutations were introduced into the specific regions of the S gene of pH120S, pIBYZS, and pYZ120S to construct the S gene mutation plasmids pH120S (I614V), pIBYZS (V617I), and pYZ120S (138−, 581−, 617−). The strategy to construct the full-length cDNA clones of IBVs are described in the schematic illustration presented in Figure 1. The genome RNAs of recombinant viruses were synthesized by T7 RNA polymerase *in vitro* and transfected into BHK-21 cells, and the recombinant viruses were rescued (Zhou et al., 2010, 2011). The recombinant viruses were propagated in allantoic cavities of 11-day-old specific-pathogen-free (SPF) embryonated chicken eggs, and allantoic fluid was collected at 40 h post infection (hpi) and stored at −80°C.

### Preparation of Primary Chicken Kidney (CK) Cells

Primary CK cells were prepared from 8-week-old chicks. Kidneys were collected, washed with phosphate buffer saline (PBS), and cut up. The resulting kidney pieces were digested with 0.25% trypsin, and 1% EDTA for 45 min at 37°C. The reaction was stopped with fetal calf serum (FCS). The cells were filtered through a sieve and collected by centrifugation at 1000 × *g* for 5 min. The kidney cells were resuspended in Medium 199 plus 3% FCS and incubated in plastic tissue flasks at 37°C with 5% CO_2_. After 48-h incubation, CK cells were ready to be used for viral infection.

### Replication Kinetics of rIBVs in Chicken Embryos

A RT-qPCR method was established based on a highly conserved area in the 5′-UTR of the IBV genome. Primers were designed according to the sequences of IBV from GenBank. The upstream primer was 5′-CCGTTGCTTGGGCTACCTAGT-3′, and the downstream primer was 5′-CGCCTACCGCTAGATGAACC-3′. The amplification product was cloned into pMD18-T (Takara) and the concentration of the plasmid was measured. A gradient dilution of 5 × 10^8^–5 × 10^2^ copies/μL was used as template for quantitation test. By plotting the cycle threshold (CT) values against the copies of the plasmid, the standard curve was generated (Jiang et al., 2020).

To examine viral replication in chicken embryos, 11-day-old embryonated SPF chicken eggs (6 eggs/group) were each inoculated with different recombinant strains at a dose of 10^7^ viral RNA copies in 100 μL. Allantoic fluids were collected from each of the 6 eggs of each group at 6-h intervals, RNA was extracted using TRIzol, and reverse transcribed into cDNA by using a Primer-Script RT Master Mix Perfect Real-Time Kit (TaKaRa, Otsu, Shiga, Japan), and RT-qPCR was performed using SYBR^®^ Premix Ex TaqTM II (TaKaRa) on an Applied Biosystems 7500 Fast Real-time PCR System.

### Replication Kinetics of rIBVs in CK Cells

CK cells were grown on 6-well plates for 48-h. Before the infection, the cells were washed with PBS. Subsequently, CK cells were inoculated with 100 μL of virus suspension containing 10^7^ viral RNA copies/well (3 wells/strain). After 60-min adsorption, the excess virus was removed by three PBS washes. Then Medium 199 was added to the infected cells. 100 μL/well viral supernatant of each group was harvested separately at different time points post-infection. Total RNA was extracted and processed as described above.

### Indirect Immunofluorescence Assay (IFA)

At 48-h post-infection, CK cells were fixed with 1 mL methanol pre-cooled at −20°C and permeabilized using 0.5% Triton X-100 in PBS. The infected cells were identified by incubation with a 1:100 dilution of serum harvested from chickens at 14 days post-injection with rIBYZ and M41 strains, followed by detection with anti-chicken IgY (IgG) (whole molecule)-FITC antibody produced in rabbit (Sigma–Aldrich, Germany; dilution 1:640). Subsequently, the nucleus was stained with 4′6-diamidino-2-phenylindole (Beyotime Biotechnology, China) for 5 min, and the immunolabeled cells were imaged using an Inverted Microscope for Industry Leica DMi8 (Leica, Germany).

### Animals and Ethics Statement

Specific pathogen-free (SPF) chicken embryos were purchased from the Beijing Merial Vital Laboratory Animal Technology Co., Ltd, China. All the animals in this study were cared for in strict accordance with the animal ethics guidelines and established protocols, and the experimental protocols were approved by the Animal Welfare and Ethical Censor Committee of the Poultry Institute, Chinese Academy of Agriculture Sciences.

### Statistical Analysis

GraphPad Prism 7 software (GraphPad Software Inc., La Jolla, CA, United States) was used for statistical analysis. In the case of replication kinetics, the data were analyzed using a two-way analysis of variance (ANOVA) to detect any significant difference between various groups.

## Results

### All Recombinant Viruses Were Successfully Rescued and Replicated Effectively in Chicken Embryos

**FIGURE 2:**
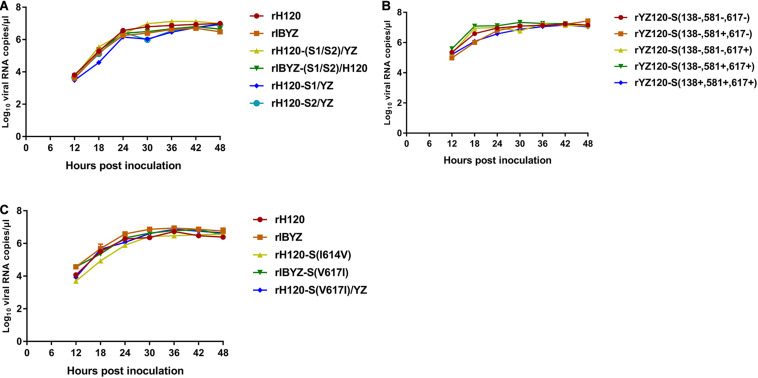
RNA replication curves of recombinants in SPF chicken embryo. Eleven-day-old embryonated SPF chicken eggs were inoculated with 100 μL containing 10^7^ viral RNA copies of **(A)** rH120, rIBYZ, rH120-(S1/S2)/YZ, rIBYZ-(S1/S2)/H120, rH120-S1/YZ and rH120-S2/YZ; **(B)** rYZ120-S(138–, 581–, 617–), rYZ120-S(138–, 581+, 617–), rYZ120-S(138–, 581–, 617+), rYZ120-S(138–, 581+, 617+) and rYZ120-S(138+, 581+, 617+); **(C)** rH120, rIBYZ, rH120-S(I614V), rIBYZ-S(V617I) and rH120-S(V617I)/YZ. The allatonic fluid was harvested at 12, 18, 24, 36, 42, and 48 h post-infection. Viral RNA copies were quantified by Real-time RT-PCR. Error bars indicate the standard deviation.

The BHK-21 cell supernatant contained viruses that were harvested at 48 h after transfection and propagated in 10-day-old SPF chicken embryos. Recombinant viruses were verified by sequencing their entire genomes. Allantoic fluid containing virus (10^7^ copies of viral RNA) was inoculated into SPF chicken embryos and harvested at different time points. All the strains could infect and proliferate in the chicken embryo and reach the peak point at 24–36 h post-infection with similar replication curves (Figure 2).

### S1/S2 Cleavage Site Is Irrelevant to CK Cell Tropism of IBV H120

**FIGURE 3:**
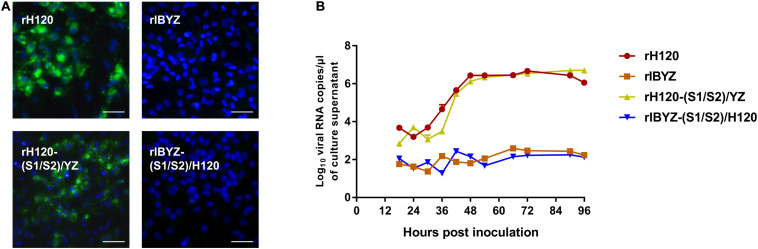
Replication characteristics of rH120-(S1/S2)/YZ and rIBYZ-(S1/S2)/H120 in CK cells. **(A)** CK cells were grown in 6-well plates for 48 h and infected with 100 μL containing 10^7^ viral RNA copies of rH120, rIBYZ, rH120-(S1/S2)/YZ, and rIBYZ-(S1/S2)/H120. After 48 h of infection, the infected cells were immunolabeled with anti-IBV serum and secondary antibody anti-chicken IgY (IgG) (whole molecule)-FITC antibody. Nuclei were labeled with DAPI (blue). Bar, 50 μm. **(B)** RNA replication curves for the recombinants in primary CK cells. CK cells in 6-well plates were inoculated with rH120, rIBYZ, rH120-(S1/S2)/YZ and rIBYZ-(S1/S2)/H120; the supernatant was harvested at 18, 24, 30, 36, 42, 48, 54, 66, 72, 90, and 96 h post-infection. Viral RNA copies were quantified by real-time RT-PCR. Y axis indicates log10 viral RNA copies/μL culture supernatant. Error bars indicate the standard deviation.

After analyzing the S gene of H120 and IBYZ strains, different motifs of the S1/S2 cleavage site were identified (H120: RRFRR_537_/S, IBYZ: HRRRR_540_/S). The S1/S2 cleavage site motifs were reciprocally expressed in the S protein of rH120 and rIBYZ strains, and the replication characteristics of the recombinants were analyzed by indirect immunofluorescence and replication curves in CK cells. Analysis of the replication kinetics in CK cells indicated that the replacement of the S1/S2 cleavage site motif with the corresponding sequence of CK cell non-adapted strain IBYZ did not alter the replication ability in CK cells. In addition, the S1/S2 cleavage site from the CK cell-adapted strain did not allow the rIBYZ-(S1/S2)/H120 strain to acquire the replication ability in CK cells (Figure 3B). The immunofluorescence analysis of CK cells infected with rH120-(S1/S2)/YZ demonstrated that the strain could infect and spread to neighboring cells, as observed for the parental virus. However, no visible green fluorescence was observed in the CK cells infected with rIBYZ-(S1/S2)/H120 strain at 48 hour post infection (hpi), as it was observed for the parental strain rIBYZ (Figure 3A). Overall, the replacement of the S1/S2 cleavage motif did not affect the ability of the parental strain to infect the CK cells.

### S2 Subunit Plays a Major Role in CK Cell Tropism of IBV H120

**FIGURE 4:**
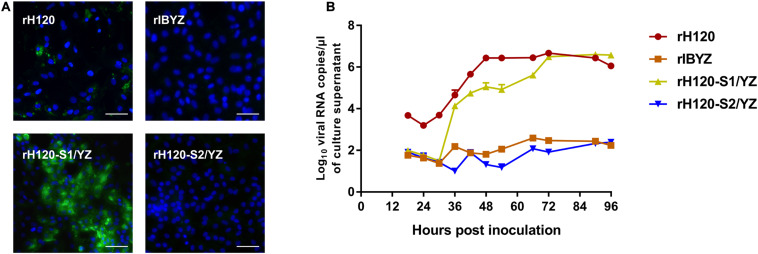
Replication characteristics of rH120-S1/YZ and rH120-S2/YZ in SPF chicken embryo and CK cells. **(A)** CK cells were grown in 6-well plates for 48 h and infected with 100 μL containing 10^7^ viral RNA copies of rH120, rIBYZ, rH120-S1/YZ, and rH120-S2/YZ. After 48 h infection, the infected cells were immunolabeled with anti-IBV serum and secondary antibody anti-chicken IgY (IgG) (whole molecule)−FITC antibody. Nuclei were labeled with DAPI (blue). Bar, 50 μm. **(B)** RNA replication curves for the recombinants in primary CK cells. CK cells in 6-well plates were inoculated with rH120, rIBYZ, rH120-S1/YZ, and rH120-S2/YZ; the supernatant was harvested at 18, 24, 30, 36, 42, 48, 54, 66, 72, 90, and 96 h post-infection. Viral RNA copies were quantified by real-time RT-PCR. *Y* axis indicates log10 viral RNA copies/μL culture supernatant. Error bars indicate the standard deviation.

Within the genomic background derived from the H120 strain, the recombinant rH120-S1/YZ or rH120-S2/YZ, with the replacement of the S1 or S2 subunit coding sequence with the corresponding sequence of CK cell non-adapted strain IBYZ, were constructed using a reverse genetic system to determine the subunit responsible for the CK cell tropism of rH120 strain. The S1/S2 cleavage site motif (RRFRR/S) from the H120 strain was retained in both recombinant strains. Immunofluorescence analysis of rH120-S1/YZ-infected CK cells at 48 hpi showed that green fluorescence was observed in some infected cells, which was similar to that of the rH120 group (Figure 4A). The apparently lower density of infected cells in the H120-inoculated cells is likely due to shedding of infected cells, as indicated by fewer nuclei. The replacement of the rH120 S2 subunit with the S2 subunit from IBYZ caused the infectivity loss in CK cells of rH120-S2/YZ. Analysis of the replication kinetics of recombinant strains in CK cells confirmed the results of IFA. The viral RNA copies of rH120-S1/YZ in CK cells increased rapidly from 30 to 36 hpi and reached a level similar to that of the parental strain rH120 at 72 hpi. Like the rIBYZ strain, rH120-S2/YZ, in which the S2 subunit was replaced with that of IBYZ strain, did not show replication in CK cells (Figure 4B). These results demonstrated that virus containing the S2 subunit of IBYZ could not infect CK cells, whereas virus containing the S2 subunit of H120 could.

### Chicken Embryo Adapted Virus YZ120 Acquires the Ability to Infect CK Cells

**FIGURE 5:**
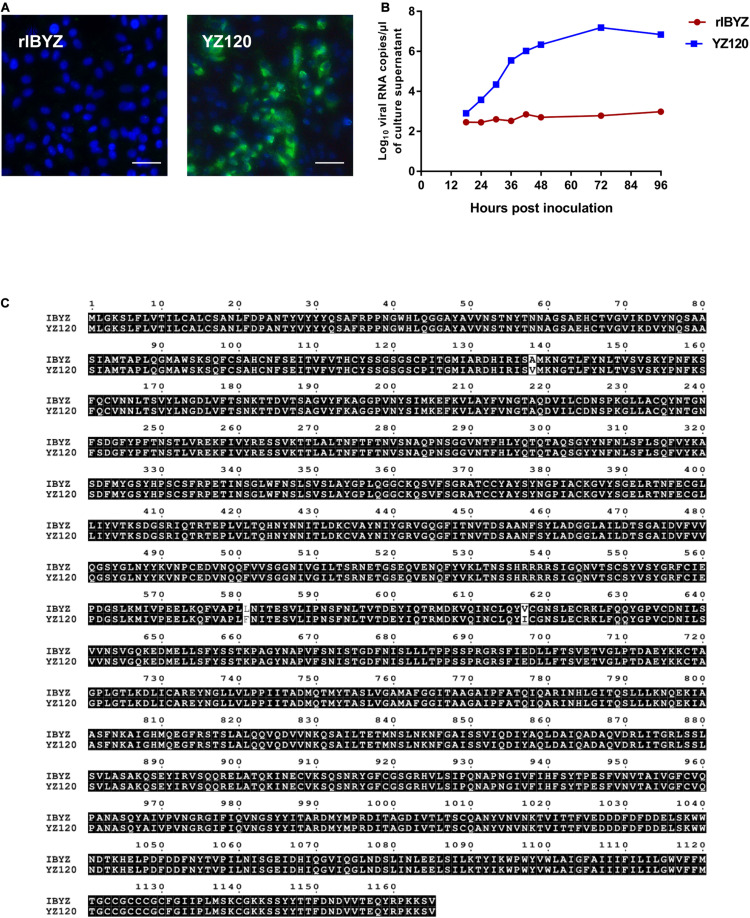
Adaptation of rYZ120 to CK cells. **(A)** CK cells were grown in 6-well plates for 48 h and infected with 100 μL containing 10^7^ viral RNA copies of rIBYZ and YZ120 strains. After 48 h infection, the infected cells were immunolabeled with anti-IBV serum and secondary antibody anti-chicken IgY (IgG) (whole molecule)- FITC antibody. Nuclei were labeled with DAPI (blue). Bars, 50 μm. **(B)** RNA replication curves for the recombinants in primary CK cells. CK cells in 6-well plates were inoculated with rIBYZ and YZ120; and the supernatant was harvested at 18, 24, 30, 36, 42, 48, 72, and 96 h post-infection. Viral RNA copies were quantified by real-time RT-PCR. Three replicates were performed, and the average was taken. *Y* axis indicates log10 viral RNA copies/μL culture supernatant. Error bars indicate the standard deviation. **(C)** Comparison of the amino acid sequences of the S protein of YZ120 and IBYZ strains. The sequences were aligned using ClustalX 2.1 and compared using Multiple Sequence Alignment by ClustalW (https://www.genome.jp/tools-bin/clustalw) and ESPript 3.0 (http://espript.ibcp.fr/ESPript/cgi-bin/ESPript.cgi). Amino acids shaded in black represent identical amino acid residues found in each sequence; non-highlighted residues represent differing amino acids.

The YZ120 strain was obtained from the parent strain IBYZ through 120 passages in chicken embryos. In contrast to IBYZ strain-inoculated cells, CK cells inoculated with the YZ120 strain exhibited high levels of infected cells 48 hpi, as indicated by immunofluorescence (Figure 5A). The results of replication kinetics showed that YZ120 strain replicates effectively in CK cells and reached the peak viral RNA copies of 10^7^ copies/μL at 72 hpi (Figure 5B). To begin to understand whether the extended tropism of YZ120 is related to its S protein, the nucleotide and predicted amino acid sequences of the S genes of IBYZ and YZ120 strains were aligned and compared using the Clustal W multiple alignment algorithm. Three nucleotide changes, each resulting in an amino acid substitution, were identified. A single nucleotide substitution, C20783T, was detected in the S1 gene of the YZ120 strain, resulting in an amino acid change, A138V. The other two nucleotide substitutions, C22111T and G22219A, occurred in the S2 gene of the YZ120 strain, resulting in amino acid changes L581F and V617I, respectively (Figure 5C).

### A138V Is Not Essential for the Invasion of YZ120 Into CK Cells

**FIGURE 6:**
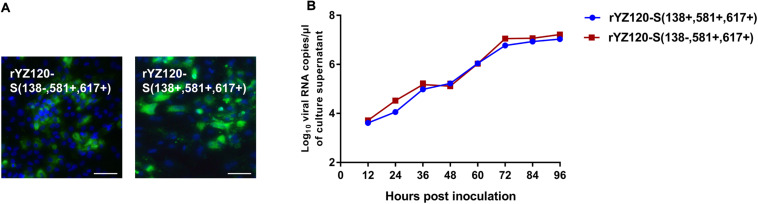
Replication characteristics of rYZ120-S(138–, 581+, 617+) and rYZ120-S(138+, 581+, 617+) in CK cells. **(A)** CK cells were grown in 6-well plates for 48 h and infected with 100 μL containing 10^7^ viral RNA copies of rYZ120-S(138–, 581+, 617+) and rYZ120-S(138+, 581+, 617+). After 48 h of infection, the infected cells were immunolabeled with anti-IBV serum and secondary antibody anti-chicken IgY (IgG) (whole molecule)-FITC antibody. Nuclei were labeled with DAPI (blue). Bar, 50 μm. **(B)** RNA replication curves for the recombinants in primary CK cells. CK cells in 6-well plates were inoculated with rYZ120-S(138–, 581+, 617+) and rYZ120-S(138+, 581+, 617+); the supernatant was harvested at 12, 24, 36, 48, 60, 72, 84, and 96 h post-infection. Viral RNA copies were quantified by real-time RT-PCR. *Y* axis indicates log10 viral RNA copies/μL culture supernatant. Error bars indicate the standard deviation.

To investigate whether one or more of the amino acids changes were required for CK cell tropism, rYZ120-S (138+, 581+, 617+), a molecular clone of CK cell-adapted strain YZ120 was generated by the reverse genetic system. Because of the results showing that the S2 subunit was the critical factor in the lack of ability of the non-adapted IBYZ strain to replicate in CK cells, we first constructed the recombinant strain rY120-S (138−, 581+, 617+), with a rYZ120 genomic backbone. One codon, encoding the amino acid residue located at position 138 in the S1 subunit was mutated to encode alanine instead of valine. Similar replication kinetics patterns were observed for rY120-S (138−, 581+, 617+) and rYZ120-S (138+, 581+, 617+) in CK cells, although the viral RNA copy numbers of the parental strain rYZ120-S (138+, 581+, 617+) were slightly lower than that of the mutation strain at time points at 24 and 72 hpi (Figure 6B). IFA confirmed the findings of the replication kinetics studies and showed similar results for the proportion of infected cells at 48 hpi, indicating that both recombinant strains were able to infect CK cells (Figure 6A). These findings demonstrated that the amino acid substitution A138V is not necessary for the invasion of YZ120 into CK cells.

### The V617I Substitution in the S Protein of rYZ120 Is Essential for Its Ability to Infect CK Cells, While L581F Also Promotes CK Cell Infection

**FIGURE 7:**
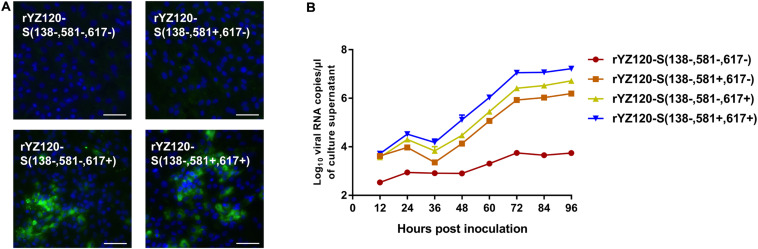
Replication characteristics of rYZ120-S(138–, 581–, 617–), rYZ120-S(138–, 581+, 617–), rYZ120-S(138–, 581–, 617+) and rYZ120-S(138–, 581+, 617+) in CK cells. **(A)** CK cells were grown in 6-well plates for 48 h and infected with 100 μL containing 10^7^ viral RNA copies of rYZ120-S(138–, 581–, 617–), rYZ120-S(138–, 581+, 617–), rYZ120-S(138–, 581–, 617+) and rYZ120-S(138–, 581+, 617+). After 48 h of infection, the infected cells were immunolabeled with anti-IBV serum and secondary antibody anti-chicken IgY (IgG) (whole molecule)-FITC antibody. Nuclei were labeled with DAPI (blue). Bar, 50 μm. **(B)** RNA replication curves for the recombinants in primary CK cells. CK cells in 6-well plates were inoculated with rYZ120-S(138–, 581–, 617–), rYZ120-S(138–, 581+, 617–), rYZ120-S(138–, 581–, 617+) and rYZ120-S(138–, 581+, 617+); the supernatant was harvested at 12, 24, 36, 48, 60, 72, 84, and 96 h post-infection. Viral RNA copies were quantified by real-time RT-PCR. *Y* axis indicates log10 viral RNA copies/μL culture supernatant. Error bars indicate the standard deviation.

In order to determine whether the other two amino acid changes are correlated to CK cell tropism, S2 gene mutation strains with one or two codon substitutions (F581L, I617V) were constructed in the rYZ120-S (138+, 581+, 617+) genomic background (Figure 1). In contrast to the rYZ120-S (138+, 581+, 617+) infected cells (Figure 6A), IFA showed no visible green fluorescence in rYZ120-S (138−, 581−, 617−) and rYZ120-S (138−, 581+, 617−) inoculated cells, indicating that the substitution of I617V led to the loss of viral infection ability in CK cells (Figure 7A). In rYZ120-S (138−, 581−, 617+) inoculated cells, only small foci of fluorescence were observed, which indicated that although F581 is not the key factor of CK cell tropism change in the YZ120 strain, substitution of F581L reduced the efficiency of CK cell infection (Figure 7A). Analysis of replication kinetics of the recombinant strains in CK cells confirmed that substitution of both F581L and I617V led to the loss of the ability of the YZ120 strain to infect primary CK cells, while the rYZ120-S (138−, 581−, 617+) strain, with a leucine residue at position 581 substituted for phenylalanine, still retains the ability to infect CK cells, but the efficiency is significantly reduced (Figure 7B). In contrast to the findings of the immunofluorescence studies, analysis of viral replication kinetics indicated that the recombinant strain rYZ120-S (138−, 581+, 617−) also had the ability to infect CK cells, although viral RNA replication was significantly lower than that in cells infected with the other two recombinant viral strains (Figure 7B). This conflicting result was explained when sequence analysis of the S gene amplified from virus in the culture supernatant of rYZ120-S (138−, 581+, 617−) infected cells showed that the codon encoding amino acid 617 of the S gene of virus recovered from the cells actually encoded isoleucine rather than valine. Thus, the rYZ120-S (138−, 581+, 617−) strain had acquired the ability to infect CK cells by mutation changing it to rYZ120-S (138−, 581+, 617+). Taken together, these results demonstrated that I617 residue in the S2 subunit may play a dominant role in extension of the tropism of the YZ120 strain to CK cells, and F581 also promotes the infection of the YZ120 strain in CK cells.

### Ability of CK Cell Infection Is Limited by I614V Substitution in rH120 S Protein

**FIGURE 8:**
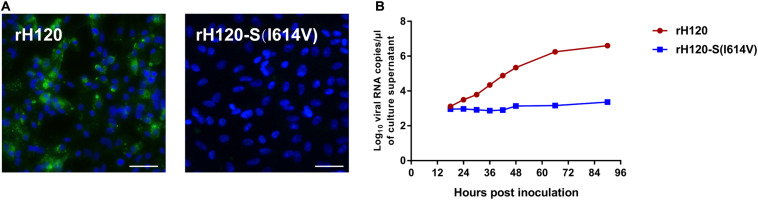
Replication characteristics of rH120 and rH120-S(I614V) in CK cells. **(A)** CK cells were grown in 6-well plates for 48 h and infected with 100 μL containing 10^7^ viral RNA copies of rH120 and rH120-S(I614V). After 48 h infection, the infected cells were immunolabeled with anti-IBV serum and secondary antibody anti-chicken IgY (IgG) (whole molecule) – FITC antibody. Nuclei were labeled with DAPI (blue). Bar, 50 μm. **(B)** RNA replication curves for the recombinants in primary CK cells. CK cells in 6-well plates were inoculated with rH120 and rH120-S(I614V); the supernatant was harvested at 18, 24, 30, 36, 42, 48, 66, 90 h post-infection. *Y* axis indicates log10 viral RNA copies/μL culture supernatant. Viral RNA copies were quantified by Real-time RT-PCR. Error bars indicate the standard deviation.

The sequence of S gene from the H120 strain was analyzed and aligned against that of the IBYZ strain using ClustalW multiple alignment algorithm. Comparison of the amino acid sequence of the S gene from H120 and IBYZ strains showed that three amino acid sites in the H120 S protein that corresponded to the substituted sites on the YZ120 S protein were A138, L578, I614, in which only residue I614 was in accordance with that on the YZ120 S protein. Therefore, the recombinant strain rH120-S (I614V), which contains the amino acid substitution I614V in the S protein, was generated using a reverse genetic system. In contrast to the parental strain rH120-infected CK cells (Figure 8A), IFA showed no visible green fluorescence in rH120-S (I614V) strain inoculated cells at 48 hpi. The results of replication kinetics also indicated that the substitution I614V in the S protein of rH120 strain induced the loss of the infection ability of the virus in CK cells (Figure 8B).

### Substitution V617I Provides IBYZ and H120-S/YZ Strains a Limited Ability to Infect CK Cells

**FIGURE 9:**
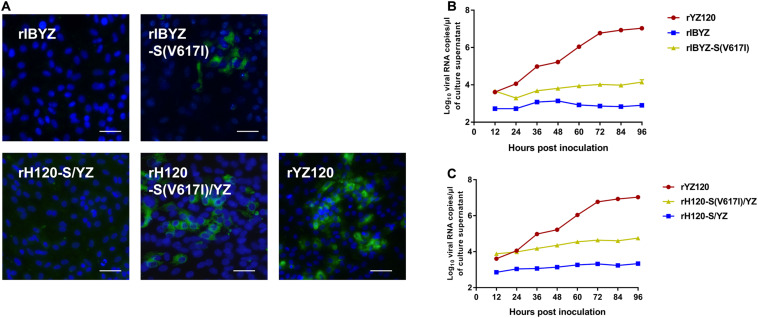
Replication characteristics of rIBYZ, rIBYZ-S (V617I), rH120-S/YZ, rH120-S (V617I)/YZ, and rYZ120 in CK cells. **(A)** CK cells were grown in 6-well plates for 48 h and infected with 100 μL containing 10^7^ viral RNA copies of rIBYZ, rIBYZ-S (V617I), rH120-S/YZ, rH120-S (V617I)/YZ, and rYZ120. After 48-h of infection, the infected cells were immunolabeled with anti-IBV serum and secondary antibody anti-chicken IgY (IgG) (whole molecule)-FITC antibody. Nuclei were labeled with DAPI (blue). Bars, 50 μm. RNA replication curves for the recombinants **(B)** rIBYZ, rIBYZ-S (V617I), and rYZ120, **(C)** rH120-S/YZ, rH120-S (V617I)/YZ, and rYZ120 in primary CK cells. CK cells in 6-well plates were inoculated with the recombinants, the supernatant was harvested at 18, 24, 30, 36, 42, 48, 66, and 90 h post-infection. Viral RNA copies were quantified by real-time RT-PCR. Y axis indicates log10 viral RNA copies/μL culture supernatant. Error bars indicate the standard deviation.

To further verify the key role of V617I in IBV CK cell tropism, we constructed the recombinant rIBYZ-S (V617I), in which an isoleucine residue was substituted for the valine residue located at position 617 of the S protein. A small number of fluorescent cells were observed 48 h after inoculation of CK cells with rIBYZ-S (V617I) (Figure 9A), and analysis of the viral RNA replication kinetics confirmed that the level of viral RNA replication was extremely low (Figure 9B). Then, the V617I substitution in the S protein was introduced into rH120-S/YZ to generate rH120-S(V617I)/YZ, and the rescued recombinant strain could effectively infect CK cells, while the infection and replication efficiency were between that of rIBYZ-S (V617I) and rYZ120 strains. Thus, the results showed that the substitution at site 617 could make IBYZ or H120-S/YZ strain acquire the ability to infect primary CK cells; however, the infection efficiency was significantly lower than that of YZ120 strain, which might be related to the differences in the genome backbone among the three strains (Figures 9A,C).

## Discussion

Previously, by replacing the S gene of rH120 and rIBYZ, we found that the S protein determines the CK cell tropism of the virus (Jiang et al., 2017), as also described previously by Casais et al. (2003). Following previous results, rH120-(S1/S2)/YZ and rIBYZ-(S1/S2)/H120, i.e., reciprocal substitution of S1/S2 cleavage site motifs in H120 and IBYZ strains were constructed, which indicated that the CK cell tropism was independent of the S1/S2 cleavage site of the H120 or IBYZ strain. Further studies demonstrated the key role of the S2 subunit in CK cell tropism of IBVs, based on the differences observed in CK cell infection ability between rH120-S1/YZ and rH120-S2/YZ. In the subsequent studies, multiple passage strain YZ120 was obtained by 120 passages in embryo, which could proliferate in CK cells. Compared to the parent strain IBYZ, three residue substitutions were found in the S protein of the YZ120 strain. Using the genomic backbone of YZ120 strain, we carried out single or multiple point substitutions on those three amino acid residues. After evaluation by IFA and proliferation curve tests of IBV-infected CK cells, we demonstrated the key role of V617I in the CK cell tropism of the YZ120 strain, which was confirmed using other recombinants, in which the amino acid encoded at S codon 617 had been changed from that in the genomic backbone of rH120 (at position 614), rH120-S/YZ, and rIBYZ strains.

Some CoVs have two cleavage sites (S1/S2 and S2’) in the S protein, which can be activated by host cell proteases in the process of infection. An S1/S2 site is located at the border between the S1 and S2 subunits, while an S2’ site is located upstream of the putative FP in the S2 subunit. The S protein of some CoVs is hydrolyzed early at the S1/S2 cleavage site by acid pH-activated serine proteases, such as furin in the trans-Golgi network of virus-producing cells (Heald-Sargent and Gallagher, 2012). In MERS-CoV, the S1/S2 cleavage site is processed during viral entry by furin (Kleine-Weber et al., 2018); however, this site is not universally required for MERS S-driven host cell entry. Similarly, “uncleavable” state by site-directed substitution of the S protein in MHV retains the virus infectivity (Gombold et al., 1993; Yamada et al., 1997). In IBVs, the cleavage of the spike protein at the S1/S2 site is not necessary for attachment but promotes the infectivity in cells (Yamada et al., 2009). rH120 strain is a molecular clone of vaccine strain H120, which was generated from the H strain isolated in 1956 by 120 embryo passages. This strain can replicate in CK cells, while the rIBYZ strain, which is a molecular clone of a QX-like IBV isolate, is unable to replicate. The S1/S2 cleavage motifs in H120 and IBYZ are RRFRRS and HRRRRS, respectively, which have the consensus R-X-[KR]-R↓motif of furin at the P4-P1 position (Tian et al., 2011). Thus, the ability of both sites to be cleaved by furin might explain why the replacement of the S1/S2 cleavage site motif from rIBYZ and rH120 strains could not change the CK cell tropism, and the difference in CK cell tropism between rIBYZ and rH120 strains is independent of the S1/S2 cleavage site motifs.

The ectodomain of all CoV spike proteins share the same organization in two domains: an NTD named S1 is responsible for binding and a C-terminal S2 domain is responsible for fusion (Belouzard et al., 2012). For some coronaviruses, including IBV, the two domains are cleaved into S1 and S2 subunits. The S1 subunit, which contains RBDs, is essential for the initial attachment of the virus (Heald-Sargent and Gallagher, 2012). Changes in the amino-terminal domain of the S1 subunit of some CoVs have been related to changes in tissue tropism *in vivo*, which might be attributed to the loss of the ability of the receptor binding (Krempl et al., 1997; Desmarets et al., 2014; Pedersen, 2014). In IBVs, α-2,3 linked sialic acids have been identified to be a receptor bound by the S1 subunit and essential for attachment of avian cells (Winter et al., 2006); however, the molecular mechanism of cell and tissue tropism is yet to be understood. Although virus binding to host cells is the first step in tropism determination that is affected by the S1 subunit (Wickramasinghe et al., 2011), S2 is responsible for membrane fusion. Sometimes, only exchanging the S1 subunit of some IBV strains may not change the tropism; the S2 subunit also affects the cell tropism of the virus. The recombinant that replaced the Beaudette strain S1 subunit with M41-CK S1 corresponding sequence replicated similarly to that of Beau-R in Vero cells, while replacing the S2 subunit of Beaudette strain with S2 subunit of M41-CK strain resulted in the loss of the ability of Beau-R strain to infect and replicate in Vero cells (Bickerton et al., 2018b). This study also indicates that the S1 subunits from both H120 and IBYZ may be able to bind to the IBV receptor, and the S2 subunit is the key factor leading to the difference of CK cell tropism between the two strains.

**FIGURE 10:**
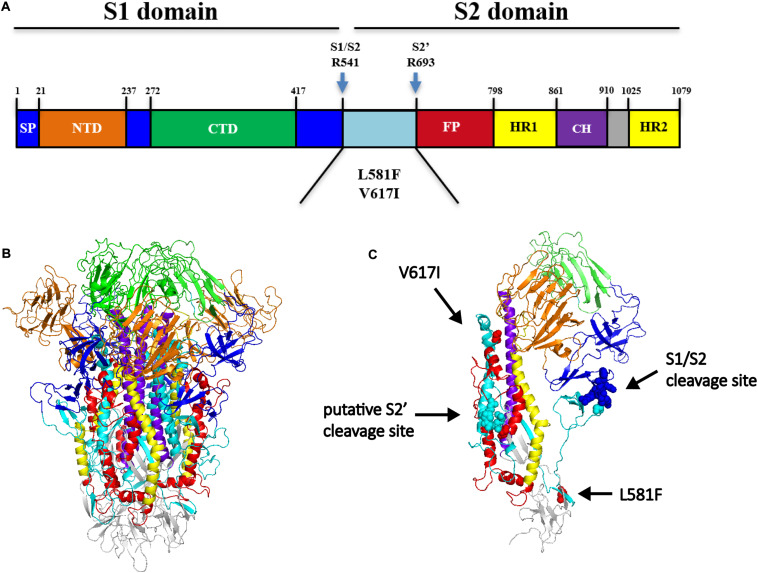
**(A)** Schematic illustration of IBYZ spike ectodomain drawn by comparing the corresponding sequence of M41 strain. NTD, N-terminal domain. CTD, C-terminal domain. FP, fusion peptide. HR1, heptad repeats 1. CH, central helix. HR2, heptad repeats 2. S2’ is a putative cleavage site, which refers to the second fusion cleavage site of the Beaudette strain. The range of FP is not determined. **(B)** Structure of IBYZ spike ectodomain in the pre-fusion conformation predicted by the Swiss-model based on the structure of the M41 S ectodomain trimer (6cv0.1). **(C)** The structure prediction of the monomer of IBYZ spike ectodomain in the prefusion conformation. The different components of Spike are colored differently, which is consistent with the schematic illustration (A). Amino acid residues at position 581 (red) and 617 (red), S1/S2 cleavage site (blue), and the putative S2’ cleavage site (cyan) are marked with spheres of different colors.

Due to the numerous sequence differences in the S2 subunits of H120 and IBYZ strains, we obtained a CK cell-adapted strain YZ120, which is a rIBYZ-derived strain continuously passaged in SPF chicken embryos, to identify the key sites on the S2 subunit related to CK cell adaptation. After sequence alignment of the S gene between YZ120 and the parental strain IBYZ, we found three amino acid substitutions, A138V in the S1 subunit and L581F and V617I in the S2 subunit. We also confirmed that A138V substitution was not related to the CK cell tropism change of the YZ120 strain, which was consistent with the conclusion that S2 determines the CK cell tropism difference between IBYZ and H120 strains. Comparing replication curves of the four mutant strains in primary CK cells, we found that rYZ120-S (138−, 581−, 617+) and rYZ120-S (138−, 581+, 617+) could effectively replicate in primary CK cells. Although the replication curve of rYZ120-S (138−, 581+, 617−) also indicated the ability to replicate in CK cells, a V617I codon mutation was found in the S gene of the virus harvested from the supernatant at 96 h post-infection. In the IFA assay, almost no fluorescence was detected in the rYZ120-S (138−, 581+, 617−)-inoculated cells, suggesting that the amino acid substitution at S position 617 might be critical for the CK cell tropism change of YZ120 strain, and L581F might promote the infection efficiency of IBVs in CK cells. Next, we analyzed the position of these two substitution amino sites, and found their localization in the region between S1/S2 cleavage and putative S2’ cleavage (Figure 10A). As a class I viral fusion protein, the S2 subunit of CoVs contains a putative FP and two heptad repeats (HR1 and HR2) (Belouzard et al., 2012). After the S1/S2 proteolytic cleavage site is activated, the conformation of S protein changed and the S2’ cleavage site and FP were exposed, which led to virus-cell fusion (Matsuyama and Taguchi, 2009; Walls et al., 2017). The analysis of the sequence of S2’ cleavage motifs in YZ120 revealed _689_SPRGR/S_694_, which could not be cleaved by furin. Nevertheless, the possibility of proteolysis by other proteases in the infectious process of susceptible chicken cells is yet to be elucidated. We predicted the structure of the S protein of the IBYZ strain, based on the cryo-electron microscopy structure of the IBV M41 S protein, by homology modeling using the SWISS-MODEL server^1^. A clove-like shape, with three S1 subunits forming a crown-like structure on top of a trimeric S2 stalk was observed in the pre-fusion conformation of IBYZ S protein (Figure 10B). The S1/S2 cleavage site and the amino acid residue at position 581 were on the periphery of the clove-like structure. In the prefusion state, the amino acid residue at position 617 of the S2 subunit, along with the putative S2’ cleavage site and FP, are located within the S1 crown-like structure (Figure 10C). At present, there is no evidence for a second cleavage site in the S protein of IBVs, except the Beaudette strain, and the exact location and size of the putative FP could not be determined. Therefore, according to the position of 617 amino acid residues, we speculate that the substitution V617I might affect the conformation of S protein in the pre-fusion state, which contributes to the exposure of the putative S2 cleavage site or FP, thereby promoting the occurrence of virus-cell fusion.

To demonstrate that the amino acid site at position 617 has the same effect in the H120 strain, we carried out a point mutation in the corresponding codon 614 of H120 S gene, which transformed the isoleucine codon into a valine codon. The mutant strain rH120-S (I614V) could not infect primary CK cells, which indicated that valine in the 614 position of S protein inhibited the invasion of IBVs into primary CK cells. To further explore whether the changes in CK cell tropism of YZ120 are determined by changes at only aa 617 in the S2 subunit, we constructed two V617I substitutions with the genomic backbone of rIBYZ or rH120-S/YZ strain. rIBYZ-S (V617I) did not acquire the ability of efficient proliferation in CK cells, while rH120-S (V617I)/YZ could infect the CK cells, its proliferation efficiency was lower than that of YZ120 strain. Based on these results, we suspect that there are other factors encoded in the backbone of the viral genome besides the S protein that can increase the replication efficiency of the virus in host cells.

We selected some of those recombinant viruses and re-tested their replication curves in primary CK cells to analyze the correlation between different recombinants and CK cell infections from the perspective of S protein and other factors (Supplementary Figure 1 and Table 1). The recombinants with the same backbone of H120 or IBYZ strain but expressing different chimeric S protein showed significant differences in the infection of primary CK cells, i.e., the order of the ability of viruses with different S proteins to infect CK cells is as follows: H120 > IBYZ-S(V617I) > IBYZ. With the same S protein from IBYZ-S (V617I), different backbones showed different virus replication efficiency in the CK cells, which indicated that there might be other factors besides S protein associated with the efficiency of IBV infection and replication in different tissues or cells. With the same S protein from IBYZ-S (V617I), different backbones showed different virus replication efficiency in the CK cells (backbone YZ120 > H120 > IBYZ), which indicated that there might be other factors besides S protein associated with the efficiency of IBV infection and replication in different tissues or cells. Thus, the substitution V617I of S protein can make YZ120 obtain the ability to infect CK cells, but the structure of this S protein has not reached the optimal evolutionary state. The effective infection in primary CK cells of YZ120 depends on the role of other structural proteins or non-structural proteins besides S protein of the genome; however, the specific mechanism is yet unclear. However, the substitution of V617I on the S protein triggered the first step of IBV virus to invade the primary CK cells, otherwise YZ120 could not enter the CK cells effectively. Therefore, the isoleucine at position 617 in the S protein from both H120 and YZ120 strains are related to CK cell tropism of IBVs, albeit the specific principle remains to be studied further.

**TABLE 1:** Analysis of infection and replication of different recombinant viruses in primary CK cells.

**Strain**	**S protein**	**Backbone**	**Infection**	**Replication**	**Conclusion**
rIBYZ-S(V617I)	IBYZ-S(V617I)	IBYZ	Yes	Low	፠ The backbone of the IBVgenome besides S gene may affect proliferation efficiency in CK cells
rH120-S(V617I)/YZ	IBYZ-S(V617I)	H120	Yes	Medium	፠ Relevant between backbone and CK cell infection:
rYZ120-S(138−, 581−, 617+)	IBYZ-S(V617I)	YZ120	Yes	High	YZ120 > H120 > IBYZ
rIBYZ	IBYZ	IBYZ	No	No	
rIBYZ-S/H120	H120	IBYZ	Yes	Medium	፠ Relevant between S protein and CK cell infection:
rIBYZ-S(V617I)	IBYZ-S(V617I)	IBYZ	Yes	Low	H120 > IBYZ-S(V617I) > IBYZ
rH120	H120	H120	Yes	High	
rH120-S/YZ	IBYZ	H120	No	No	፠ Relevant between S protein and CK cell infection:
rH120-S(V617I)/YZ	IBYZ-S(V617I)	H120	Yes	Medium	H120 > IBYZ-S(V617I) > IBYZ
rH120-S/YZ	IBYZ	H120	No	No	፠ S protein but not backbone of IBVs is a determinant of CK cell tropism. ፠S protein of IBYZ strain does not provide the ability to infect CK cells.
rIBYZ	IBYZ	IBYZ	No	No	
rYZ120-S(138−, 581−, 617−)	IBYZ	YZ120	No	No	

## Conclusion

The S2 subunit is the determinant factor of the difference in CK cell tropism between H120 and IBYZ strains. The adaptation of the YZ120 strain to CK cells is independent of the A138V substitution, while V617I substitution leads to the CK cell tropism changes in the YZ120 strain, and L581F promotes the infectivity of the YZ120 in CK cells. In addition, there could also be other factors in the genomic backbone of IBVs associated with the CK cell tropism, which need further investigation.

## Data Availability Statement

All data, models generated or used during the study are available from the corresponding author by request.

## Author Contributions

YJ and SZ designed and conducted the experiments. YJ, SZ, XC, and YY performed the experiments. YJ, MG, XS, and JL analyzed and interpreted the data. YJ and SZ wrote and revised the manuscript. All the authors contributed to the article and approved the submitted version.

## Conflict of Interest

The authors declare that the research was conducted in the absence of any commercial or financial relationships that could be construed as a potential conflict of interest.

## Footnotes

1 https://swissmodel.expasy.org/interactive

## Supplementary Material

Supplementary Figure 1 Replication characteristics of rIBYZ-S (V617I), rH120-S (V617I)/YZ, rYZ120-S (138−, 581−, 617+), rIBYZ, rIBYZ-S/H120, rH120, rH120-S/YZ, in CK cells. **(A)** rIBYZ-S(V617I), rH120-S (V617I)/YZ, rYZ120-S (138−, 581−, 617+), **(B)** rIBYZ, rIBYZ-S/H120, rIBYZ-S (V617I), **(C)** rH120, rH120-S/YZ, rH120-S (V617I)/YZ in primary CK cells. CK cells in 6-well plates were inoculated with the recombinants, the supernatant was harvested at 12, 24, 36, 48, 60, 72, 84, and 96 h post-infection. Viral RNA copies were quantified by real-time RT-PCR. Y axis indicates log10 viral RNA copies/μL culture supernatant. Error bars indicate the standard deviation.
