# Supplementary figures and images for: The V617I Substitution in Avian Coronavirus IBV Spike Protein Plays a Crucial Role in Adaptation to Primary Chicken Kidney Cells
Source: Front Microbiol. 2020 Dec 18;11:604335. doi: 10.3389/fmicb.2020.604335 (PMC7775488; doi:10.3389/fmicb.2020.604335)

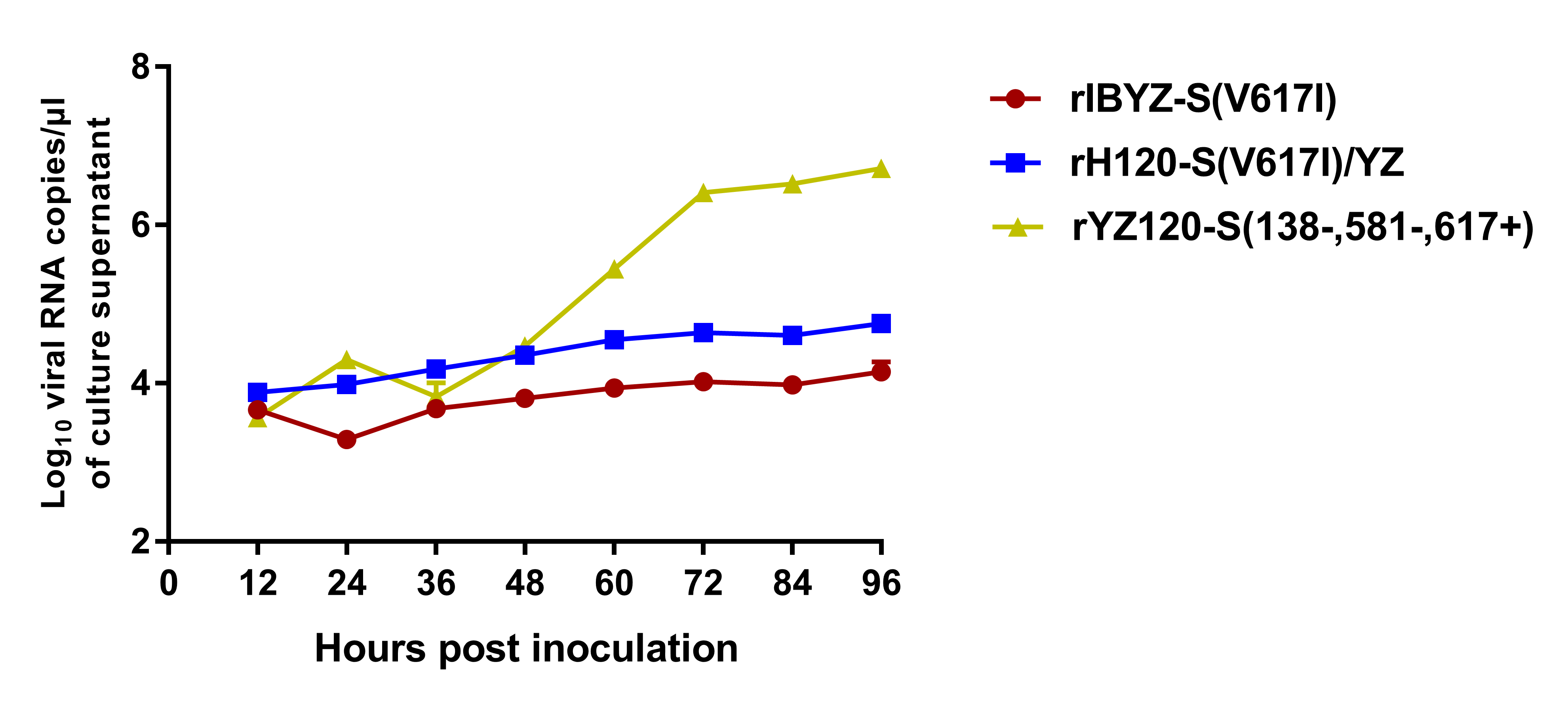

Supplement: Supplementary Figure 1 — Replication characteristics of rIBYZ-S (V617I), rH120-S (V617I)/YZ, rYZ120-S (138−, 581−, 617+), rIBYZ, rIBYZ-S/H120, rH120, rH120-S/YZ, in CK cells. (A) rIBYZ-S(V617I), rH120-S (V617I)/YZ, rYZ120-S (138−, 581−, 617+), (B) rIBYZ, rIBYZ-S/H120, rIBYZ-S (V617I), (C) rH120, rH120-S/YZ, rH120-S (V617I)/YZ in primary CK cells. CK cells in 6-well plates were inoculated with the recombinants, the supernatant was harvested at 12, 24, 36, 48, 60, 72, 84, and 96 h post-infection. Viral RNA copies were quantified by real-time RT-PCR. Y axis indicates log10 viral RNA copies/μL culture supernatant. Error bars indicate the standard deviation. [file Image_1.tif]

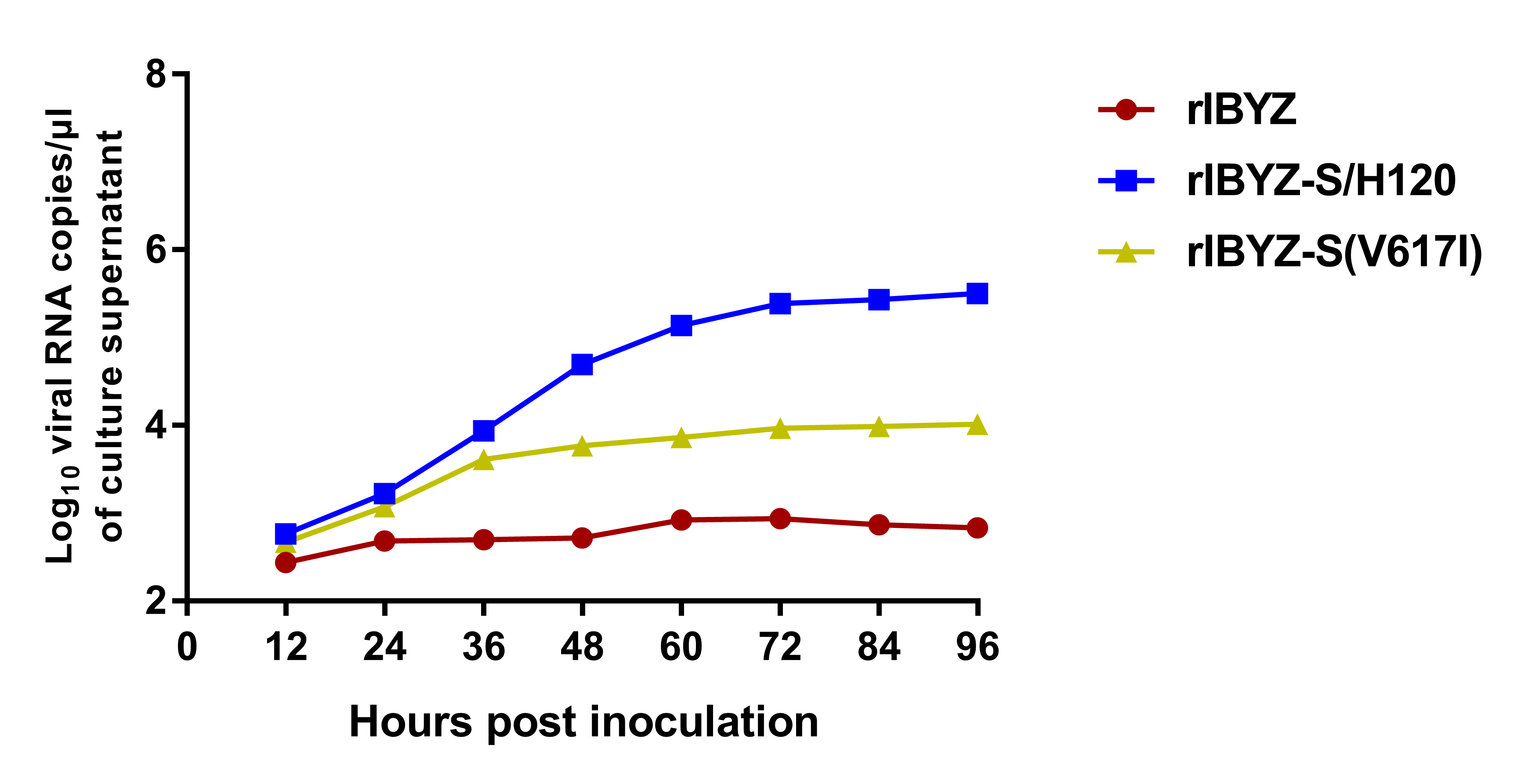

Supplement: Supplementary file 3 [file Image_2.tif]

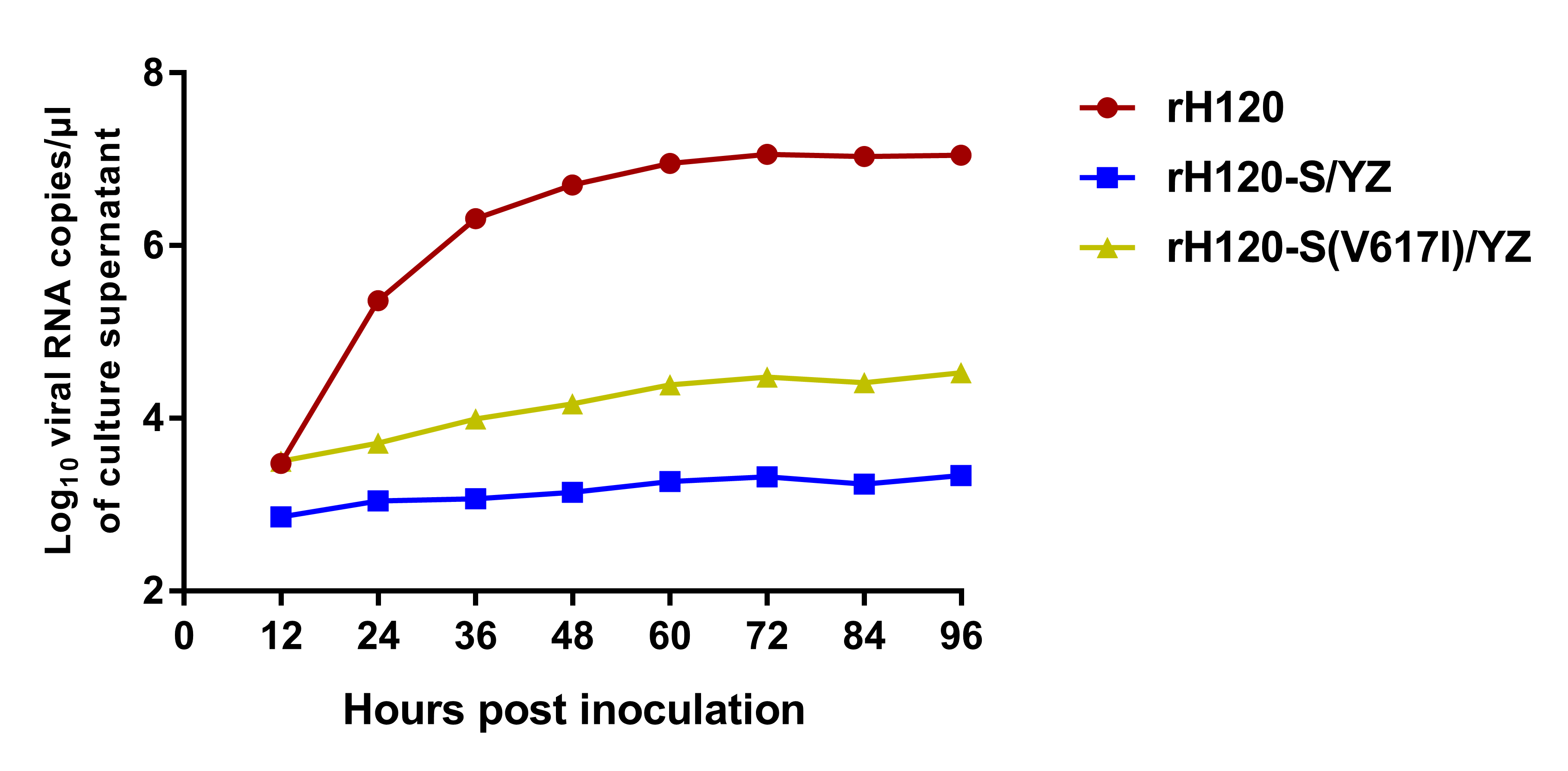

Supplement: Supplementary file 4 [file Image_3.tif]
